# Effects of Optical Turbulence and Density Gradients on Particle Image Velocimetry

**DOI:** 10.1038/s41598-020-58077-5

**Published:** 2020-02-07

**Authors:** Silvia Matt, Gero Nootz, Samuel Hellman, Weilin Hou

**Affiliations:** 1 Ocean Sciences Division, US Naval Research Laboratory, Stennis Space Center, MS 39529 USA; 2 Oceanography Division, NRC Research Associate at US Naval Research Laboratory, Stennis Space Center, MS 39529 USA; 3 Dantec Dynamics Inc., Holtsville, NY 11742 USA; 4 Present Address: Department of Marine Science, University of Southern Mississippi, Stennis Space Center, MS 39529 USA; 5 Present Address: Department of Medical Physics, Memorial Sloan Kettering Cancer Center, New York, NY 10065 USA

**Keywords:** Physical oceanography, Fluid dynamics

## Abstract

Particle image velocimetry (PIV) is a well-established tool to collect high-resolution velocity and turbulence data in the laboratory, in both air and water. Laboratory experiments are often performed under conditions of constant temperature or salinity or in flows with only small gradients of these properties. At larger temperature or salinity variations, the changes in the index of refraction of water or air due to turbulent microstructure can lead to so-called optical turbulence. We observed a marked influence of optical turbulence on particle imaging in PIV. The effect of index of refraction variations on PIV has been described in air for high Mach number flows, but in such cases the distortion is directional. No such effect has previously been reported for conditions of isotropic optical turbulence in water. We investigated the effect of optical turbulence on PIV imaging in a large Rayleigh-Bénard tank for various path lengths and turbulence strengths. The results show that optical turbulence can significantly affect PIV measurements. Depending on the strength of the optical turbulence and path length, the impact can be mitigated in post-processing, which may reduce noise and recover the mean velocity signal, but leads to the loss of the high-frequency turbulence signal.

## Introduction

Optical turbulence refers to the impact of index of refraction variations on optical signal transmission^1^. In the atmosphere, optical turbulence is well-studied and responsible for phenomena such as the twinkling of stars in the night sky. Less is known about oceanic optical turbulence, in part due to the difficulties encountered when attempting to measure underwater optical turbulence in its natural environment in oceans and lakes^2–5^. Such optical turbulence can be pronounced in the oceanic thermocline, in areas where precipitation leads to freshwater lenses on the ocean surface, or under an ice-covered sea surface.

**Figure 1:**
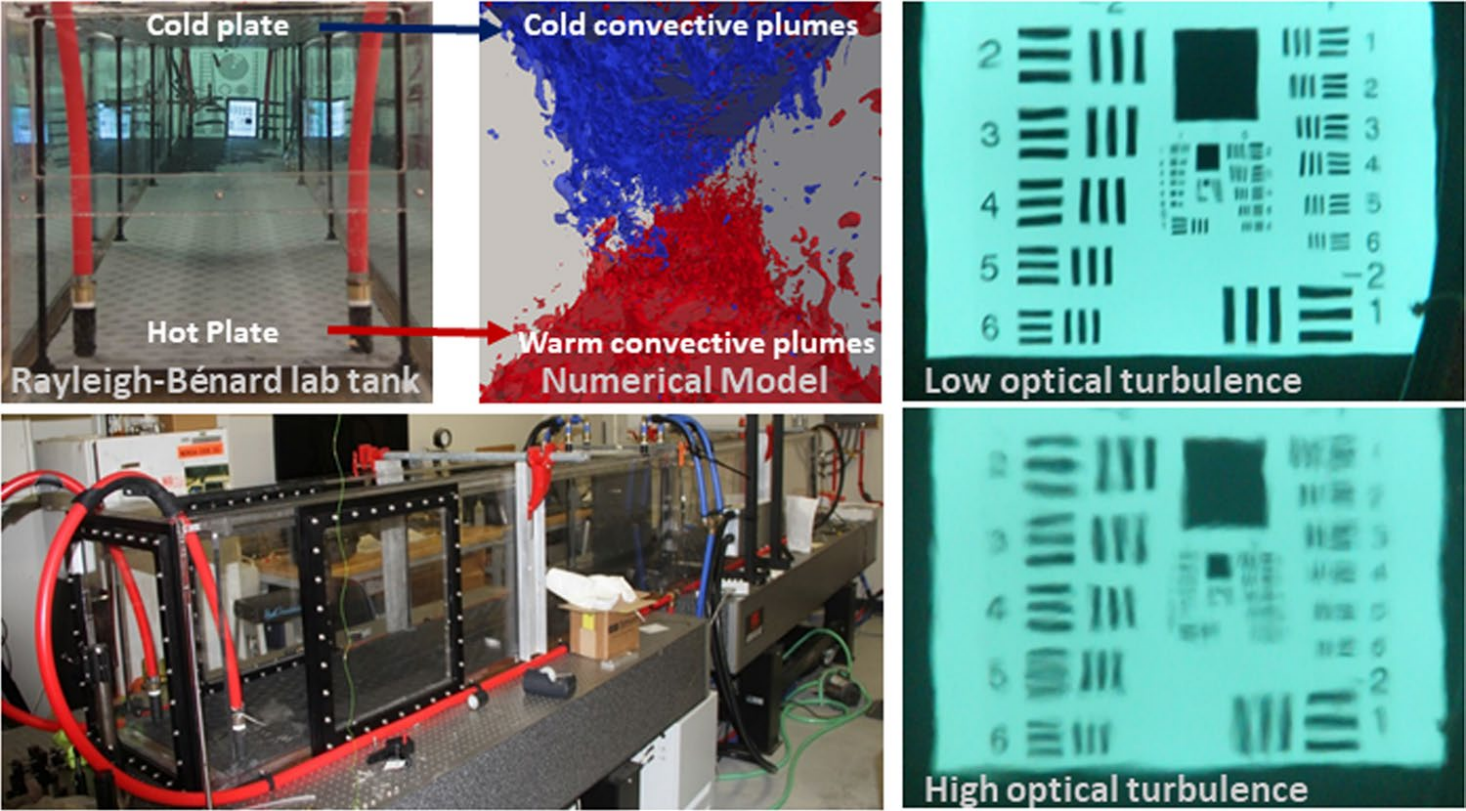
Unique Simulated Turbulence and Turbidity Environment (SiTTE) designed to generate convective turbulence at various turbulence intensities. Top: View into laboratory tank at NRLSSC (left), same view into CFD numerical tank (center) and video still of optical chart across the 5 m long tank at low optical turbulence (right). Bottom: Side view of 5 m long tank showing optical windows (left) and video still of optical chart across the 5 m long tank at highest optical turbulence (right).

The Simulated Turbulence and Turbidity Environment (SiTTE) at the Naval Research Laboratory Stennis Space Center (NRLSSC), consists of a large Rayleigh-Bénard laboratory tank that allows the generation of convective turbulence at controlled turbulence intensity, which allows the study of optical turbulence in a controlled laboratory setting (Fig. 1). This controlled turbulence environment is complemented by computational fluid dynamics (CFD) simulations and has previously been described in detail in^6^. The unique combination of laboratory environment and complementary numerical model offer a well-quantified framework for controlled repeatable experiments, which has provided a test bed for novel optical sensor technologies, as well as the use of adaptive optics and other methods to mitigate turbulence effect on optics^7–9^.

During the investigation of velocity fields and optical turbulence in SiTTE using particle image velocimetry (PIV), an established tool to study laboratory scale fluid flow, we noted a pronounced effect of the optical turbulence, in particular for high optical turbulence and long optical path, on particle imaging in PIV. The optical turbulence distorts the particles in the PIV images and gives them a “shooting star”-like appearance. While the effect of optical inhomogeneity on PIV has been reported in compressible gas flows^10,11^, these distortions are typically geometrically oriented (e.g. supersonic shocks) as opposed to the stochastic distribution of stratified fluid cells observed in the present study. With the increasing interest in using PIV in the field in ocean and lakes, away from the uniform density conditions encountered in a controlled laboratory setting, the impact of turbulent density fluctuations on the imaging and resulting velocity fields can no longer be neglected. The optical manifestations of turbulence and physical microstructure are known to affect *in-situ* imaging systems, such as the shadowgraph imagers used in the biological oceanography community to collect high-resolution images of zooplankton and micronekton. There, the density variations due to optical turbulence are typically treated as background distortions and marked optical turbulence has been reported in regions of strong thermoclines and haloclines, such as those found near oceanic fronts^12^, in near-surface buoyancy-driven currents, including riverine outflow and rain/freshwater lenses, or in underground aquifers. Optical turbulence in thermoclines in lake and ocean have been described in^3–5^, respectively. The impact from density variations is expected to be equally pronounced under ice, an emerging area of interest for wave and turbulence studies of wide-ranging importance for local and global forecasting models, with numerous projected applications for PIV measurements. With such marked effects of density variations on optical imaging, the impact on PIV imaging and on inferred velocity fields warrants a thorough investigation. We investigated the effect and how to moderate the impact on the derived velocity fields by performing experiments at varying optical turbulence strengths and different optical path lengths.

The SiTTE laboratory tank is 5 m long with a cross section of 0.5 m by 0.5 m. The convective turbulence is driven by water-heated and -cooled aluminum plates at the tank bottom and top, respectively. This allows for the generation of different strengths of convective turbulence, with Rayleigh numbers ranging from 1·10^10^ to 4.5·10^10^. A complementary computational fluid dynamics (CFD) Large–Eddy Simulation (LES) model emulating the turbulence tank completes the laboratory setup.

The effect of optical turbulence on PIV was studied in the convective tank for a range of turbulence levels, from low optical turbulence, with a temperature difference between the bottom and top plates ΔT ≈ 4 °C and Rayleigh number Ra = 1·10^10^, to high optical turbulence, with Rayleigh number Ra = 3·10^10^ and ΔT ≈ 13 °C. Figure 1 (top right-most panel) shows the effect of low and high optical turbulence on the image of an optical chart recorded across 5 m path (the length of the tank) in filtered tap water. A detailed characterization and calibration of the turbulence tank in terms of turbulence characteristics and optical turbulence conditions has been reported in^6,13^, respectively. Thus, the respective turbulence strengths can be related to the turbulent kinetic energy dissipation rate ε and temperature variance dissipation rate χ, as well as the index of refraction structure constant C_n_^2^, a measure of optical turbulence strength. For Ra = 1·10^10^, our “low” optical turbulence conditions, the turbulent kinetic energy dissipation rate ε was estimated in^6^ at around ε = 3·10^−8^ W/kg, for the “high” turbulence conditions at close to ε = 4·10^−7^ W/kg. The temperature variance dissipation rate χ was reported as χ = 1·10^−4^ C^2^/s for “low”, and χ = 2·10^−3^ C^2^/s for “high” turbulence. The details of these measurements reveal the distinction between turbulence regimes to be more pronounced in temperature rather than velocity, which can be related to the convective forcing driving the turbulence and the relatively benign velocities in the flow. The details of the turbulence dynamics in the tank are beyond the scope of this paper and the interested reader is referred to^6^. Expanding on the turbulence measurements and numerical model presented in^6^, the optical turbulence environment in the convective tank was described in^13^ and the range of convective conditions was put into context of the index of refraction structure constant C_n_^2^. The optical turbulence conditions reported here then correspond to about C_n_^2^ = O(10^−10^) m^−2/3^ for “low” and C_n_^2^ = O(10^−9^) m^−2/3^ for “high” turbulence conditions. Since the impact on imaging is a function of optical path length, the Rytov variance for the respective optical path lengths and convective conditions is a better predictor of optical degradation. The Rytov variance (unitless) for a plane wave is defined as σ^2^ = 1.23 C_n_^2^ *k* ^7/6^ L ^11/6^ (where k = 2π/λ is the optical wave number, λ the wavelength, and L is the propagation path length) and is given in Table 1 for our range of experiments. Note that a Rytov variance σ^2^ > 0.3 is considered a transition from the low to moderate optical turbulence regime.

**Table 1:** Experimental matrix showing the Rytov variance, a measure of optical turbulence strength, for the respective convective turbulence level and path lengths of the experiments presented in this paper.

	Low convective turbulence: 4 degrees	Intermediate convective turbulence: 8 degrees	High convective turbulence: 13 degrees
Nearfield: 0.5 m	0.01	0.02	0.04
Midfield: 1.2 m	0.02	0.08	0.18
FarMidfield: 1.6 m	0.04; no data	0.13	0.31
Farfield: 1.9 m	0.06	0.18	0.43

Note that the low optical turbulence, FarMidfield data is missing, due to a problem with the measurement.

## Results

**Figure 2:**
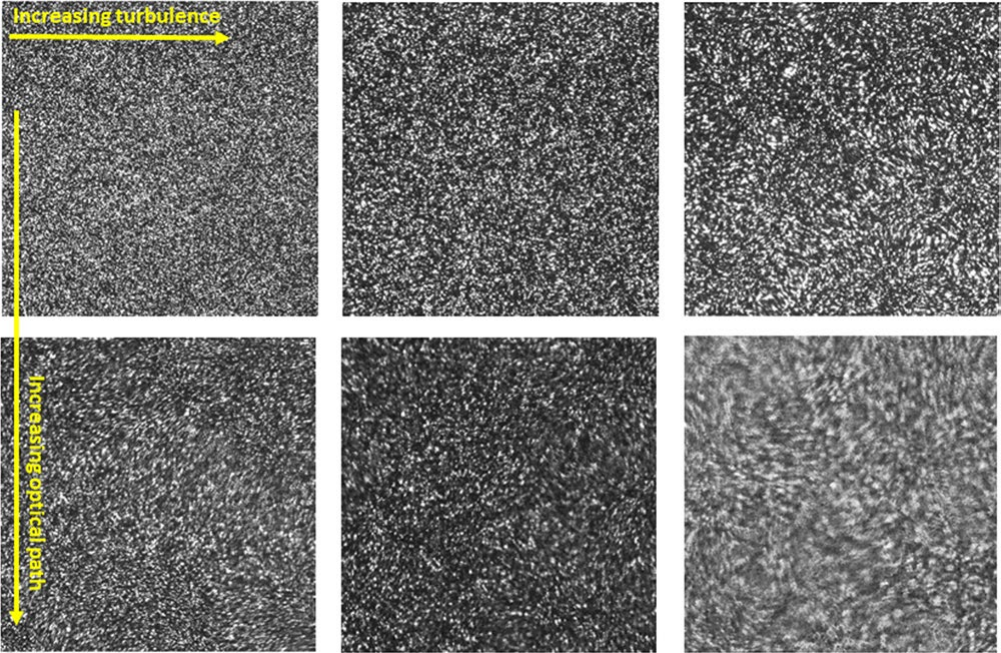
Raw images from PIV show the distortion in the raw image of illuminated PIV particles for varying intensities of optical turbulence at different optical path lengths. Optical turbulence is increasing from left to right and images are for ΔT = 4 °C, ΔT = 8 °C, ΔT = 13 °C, and path length is L = 0.5 m for the top row, and L = 2 m for the bottom row.

The image degradation in PIV was investigated for different optical turbulence strengths and path lengths ranging from 0.5 m to 1.9 m, almost half-way across the tank length of 5 m. In all cases, the field of view (FOV) and resulting image magnification were kept constant to allow a direct comparison of results. In order to keep the magnification constant, the total effective path length (via air outside the tank and water in the tank) remained constant, however, the path lengths referred to in this paper are the path lengths through water in the tank. In this paper, we refer to the experiment with the shortest optical path by “Nearfield” and the case with the longest optical path as “Farfield”, with intermediate cases referred to as “Midfield” and “FarMidfield” as shown in Table 1. Figure 2 illustrates the distortion observed in the PIV raw images due to optical turbulence and the varying impact depending on optical path length and turbulence intensity. A Laser Doppler Anemometer (LDA) system was used to provide an independent reference measurement of velocity. The instrument, which provides a point measurement of velocity, was positioned in such a way that the measurement point was in plane with the PIV laser sheet.

**Figure 3:**
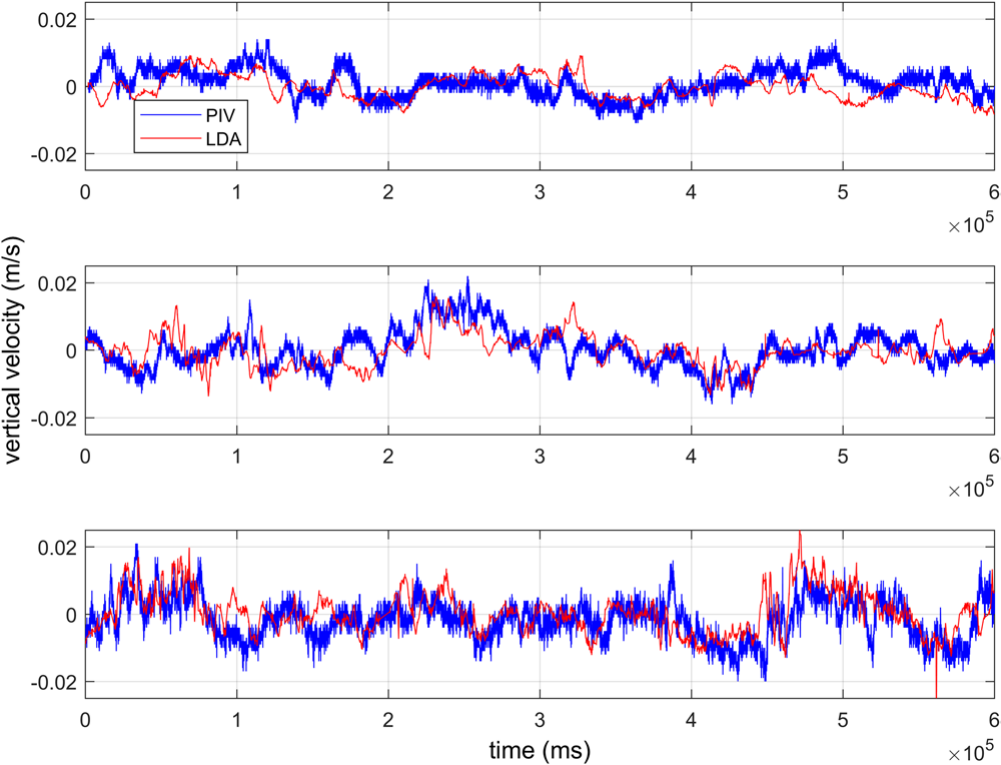
Time series of Adaptive Correlation PIV (at location of LDA beam crossing) and LDA velocity for the Nearfield measurements. Top panel show the vertical velocity at low optical turbulence, ΔT = 4 °C. Middle panel is for intermediate optical turbulence, ΔT = 8 °C. Bottom panel shows high optical turbulence, ΔT = 13 °C.

**Figure 4:**
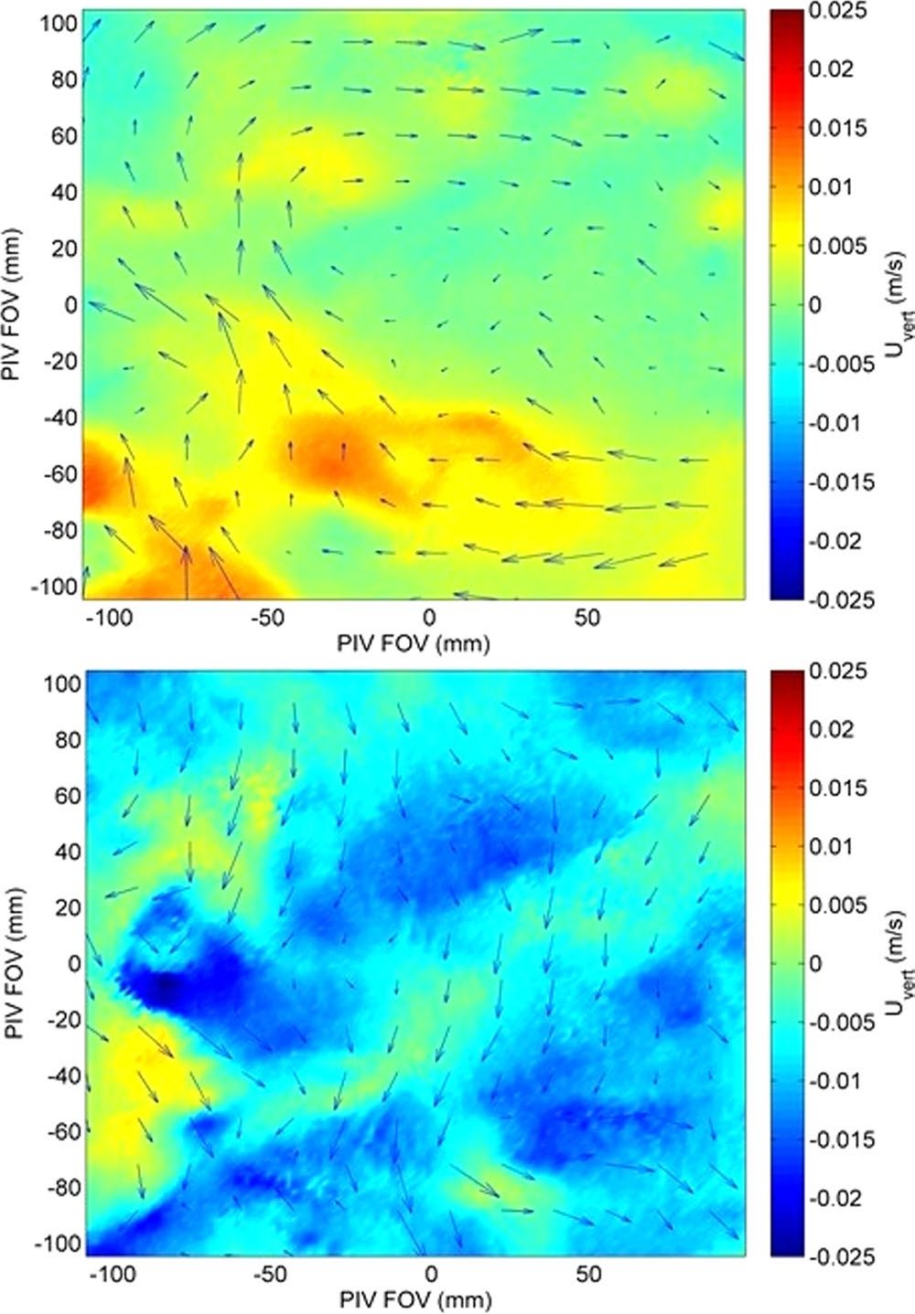
Instantaneous velocity fields from Adaptive Correlation PIV results for the Nearfield case, path length 0.5 m. Top panel show the vertical velocity at low optical turbulence, ΔT = 4 °C. The velocity for high optical turbulence, ΔT = 13 °C, using the same post-processing algorithm is shown on the bottom and reveals considerable noise. Color scale is in m/s and the arrows indicate velocity vectors.

A comparison between the time series of PIV velocity results, processed using the Adaptive Correlation algorithm, at the location of the LDA beam crossing (the LDA measurement location) and the LDA velocity for the Nearfield measurements for low optical turbulence (ΔT = 4 °C), intermediate optical turbulence (ΔT = 8 °C), and high optical turbulence (ΔT = 13 °C) show that the velocity signals agree well in terms of velocity magnitude and scale of variability (Fig. 3). The slight lag between the signals suggests that the alignment between the two measurement points was slightly offset, possibly positioning the two measurement locations at opposing points in a turbulent overturn. However, overall magnitude and signal variability are consistent. Contour plots of velocity from the corresponding PIV measurement plane for two instantaneous snap shots at low and high turbulence show that the velocity field is well resolved, without excessive noise in the PIV data (Fig. 4, top). The velocity magnitudes and flow characteristics are consistent with the velocity fields previously reported from the SiTTE tank under low optical turbulence conditions as described in^6^.

Since the degrading effect of optical turbulence on optical signal transmission is cumulative over the length of the optical path, the strongest impact can be anticipated for the high optical turbulence in the Farfield case. This was confirmed qualitatively in the raw images acquired by PIV, the most blurring and distortion occurs for high optical turbulence at long optical path lengths (Fig. 2). The velocity data confirmed this effect as seen in Figs. 4 (bottom) and 5 (top), which show excessive noise in the PIV velocity for highest optical turbulence, at the Farfield measurement location when using the same post-processing as used for the Nearfield data, i.e. a fixed interrogation area of 16 × 16 pixels.

**Figure 5:**
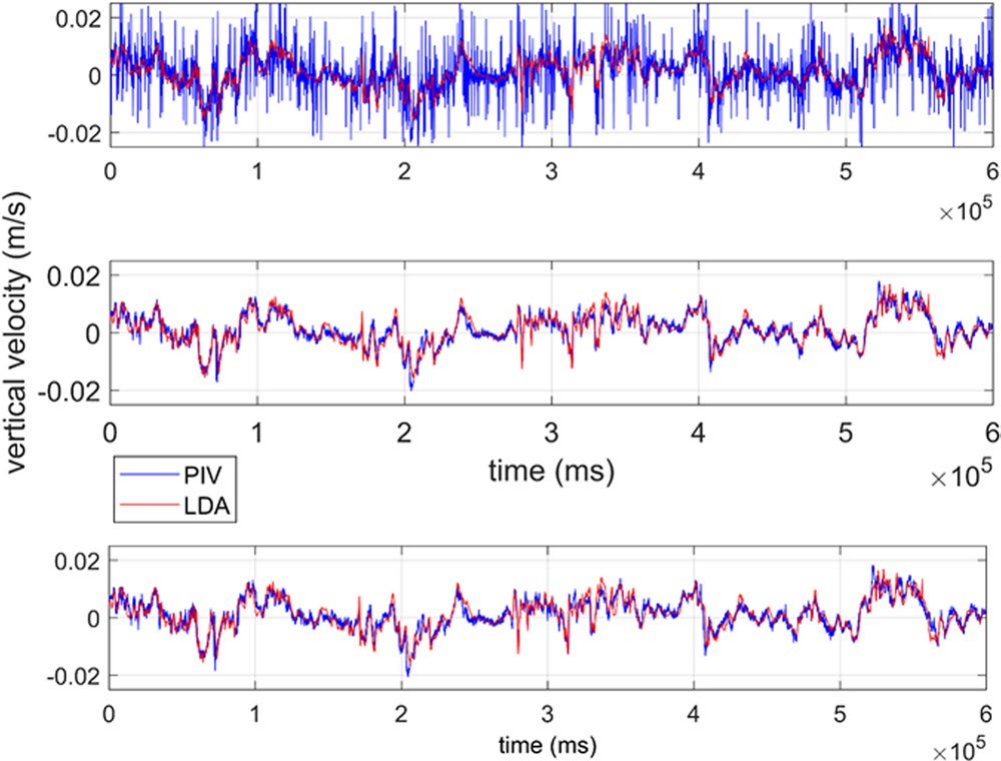
Time series of PIV results at the location of LDA beam crossing using Adaptive Correlation and Adaptive PIV algorithms for the Farfield measurements with a path of close to 2 m. Top panel shows the vertical velocity inferred from Adaptive Correlation using a fixed interrogation area of 16 × 16 pixels at highest optical turbulence, ΔT = 13 °C. There is significant noise in the PIV signal, due to the optical turbulence effect of smearing and defocusing the PIV particles. Middle and bottom panels are also for highest optical turbulence, ΔT = 13 °C, but using a fixed interrogation area of 64 × 64 pixel for the middle panel and, in the bottom panel, inferred with the Adaptive PIV algorithm, which varies the interrogation area depending on particle concentration and velocity gradients.

**Figure 6:**
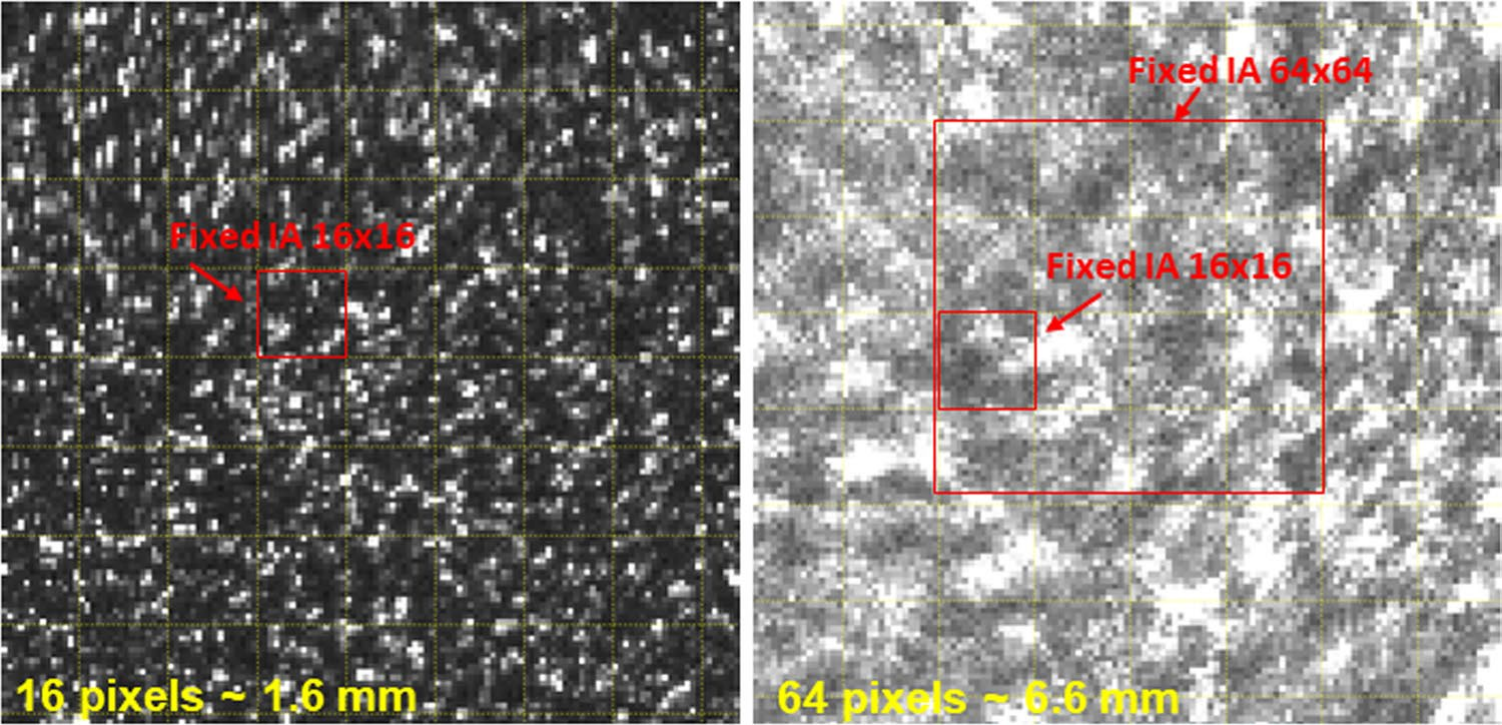
Zoomed PIV raw image with a 16 × 16 pixel grid overlaid in yellow shows that the high optical turbulence distorts the particle signatures in the image and this affects how many particles are resolved within the interrogation area. Left is at low optical turbulence, ΔT = 4 °C, Nearfield, right is for high optical turbulence, ΔT = 13 °C, Farfield.

This excessive noise is due to the blurring effect of optical turbulence on the PIV raw images, as illustrated in Fig. 6. Figure 6 shows zoomed-in regions of raw PIV images acquired for the Farfield measurement plane with a 16 × 16 pixel grid overlaid. It is apparent in the high turbulence case that the particle boundaries inside the PIV interrogation area are blurred and not enough individual particles are visible inside the 16 × 16 pixel box. This lack of particle resolution can lead to failure of the post-processing algorithm, when calculating velocities from blurred raw images using a fixed interrogation area size. This effect can be mitigated by choosing an Adaptive PIV algorithm that optimizes the interrogation area size depending on particle density and thus image clarity, which comes at the expense of spatial resolution of the resulting velocity field.

After reprocessing the Farfield data by either increasing the interrogation area to a fixed 64 × 64 pixels, or by processing with the Adaptive PIV algorithm, which allows the interrogation area to vary in size, the noise in the PIV data is largely removed and the match between PIV and LDA velocity is excellent (Fig. 5, middle and bottom; for the remainder of this paper, when referring to Farfield PIV data, we refer to the velocities calculated with the appropriate post-processing, i.e., the Adaptive PIV algorithm with the settings mentioned below in Methods). However, the velocity time series shows that the small-scale variability in the signal is reduced, reminiscent of a low-pass filter. Here, the loss of spatial resolution is reflected in the loss of high-frequency variability in the time series data. Instantaneous velocity contour plots (not shown) confirm this effect, which is the result of the Adaptive PIV algorithm locally increasing the interrogation area size as needed to allow correlation. Thus, while careful and appropriate post-processing allows to recover the correct low-frequency velocity signal from the distorted PIV images, the removal of the noise also affects the signal resolution, and we effectively lose local information on the turbulence signal in the flow.

Table 2 further illustrates this by reporting the variance calculated from the velocity time series. Here, the variance is defined as

1 $$V=\frac{1}{N-1}\mathop{\sum }\limits_{i=1}^{N}|{A}_{i}-\mu {|}^{2}$$

where μ is the mean of a random variable A, N is the number of observations, and

2 $$\mu =\frac{1}{N}\mathop{\sum }\limits_{i=1}^{N}{A}_{i}$$

**Table 2:** Velocity variance (in m^2^/s^2^), a proxy for turbulent fluctuations, from PIV and LDA is shown for different turbulence strengths and path lengths.

	Low optical turbulence: 4 degrees	Intermediate optical turbulence: 8 degrees	High optical turbulence: 13 degrees
Nearfield PIV 0.5 m	1.731e-05	3.281e-05	4.217e-05
Nearfield LDA 0.5 m	1.646e-05	3.343e-05	3.793e-05
Midfield PIV 1.2 m	2.298e-05	4.308e-05	4.967e-05
Midfield LDA 1.2 m	2.571e-05	7.220e-05	6.520e-05
FarMidfield PIV 1.6 m	No data	3.111e-05	6.950e-05
FarMidfield LDA 1.6 m	1.788e-05	3.479e-05	9.903e-05
Farfield PIV 1.9 m	2.250e-05	2.360e-05	2.886e-05
Farfield LDA 1.9 m	3.056e-05	3.651e-05	4.078e-05

The variance can provide information on the velocity variations around a mean and can thus be considered a proxy for turbulent fluctuations or the turbulent kinetic energy (TKE) as defined in (3). Here, *u*, *v*, and *w* are the components of velocity and the overbar represents the mean as in (2). Note that in this paper, we only report on the vertical velocity component *v*, justified by the isotropic nature of the turbulence and due to the single-component reference velocity generated by the LDA system.

3 $$TKE=\frac{1}{2}(\overline{{(u-\bar{u})}^{2}}+\overline{{(v-\bar{v})}^{2}}+\overline{{(w-\bar{w})}^{2}})$$

From (1) and (3), the physical analogy between velocity variance and turbulent kinetic energy becomes readily apparent.

Taking into account measurement uncertainties, PIV and LDA agree very well for the Nearfield location for three different turbulence strengths. However, for longer optical path lengths, especially at high optical turbulence, the PIV measurements processed with variable, optimized interrogation area size, show a variance that is too low when compared to the reference velocity collected with the LDA. This is consistent with the velocity signal shown in the time series in Fig. 5, where the high-frequency variability is lost after processing with an appropriate interrogation area size.

**Figure 7:**
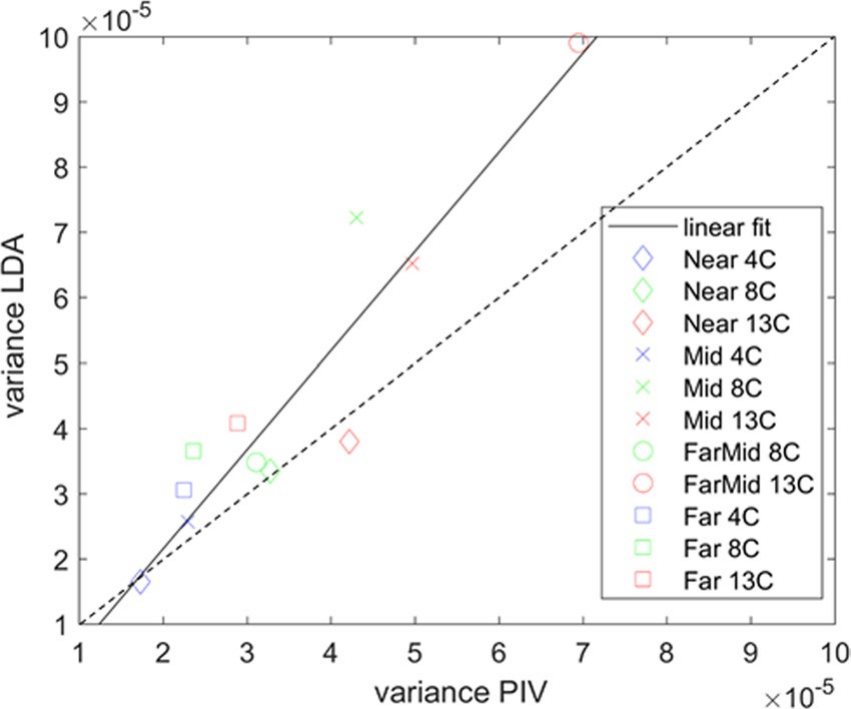
Plot of the data in Table 2, variance (in m^2^/s^2^) of PIV data against the variance from the LDA velocities.

The data in Table 2 is represented graphically in Fig. 7, where the variance from the PIV velocities is plotted against the variance from LDA velocities. For short optical path and low turbulence strength, the data fall close to a line with slope 1. For higher turbulence and longer optical paths, the PIV consistently underestimates the signal variance, when compared to the variance of the reference velocity from LDA. This is consistent with the loss of high-frequency information due to a decrease in spatial resolution. This effect becomes increasingly apparent for optical turbulence strengths represented by a Rytov variance σ^2^ > 0.05, which in our setup represents the intermediate convective strength and higher, and the path lengths of about 1 m and up. Rytov variances up to σ^2^ = 0.23 have been reported in the oceanic thermocline^5^.

**Figure 8:**
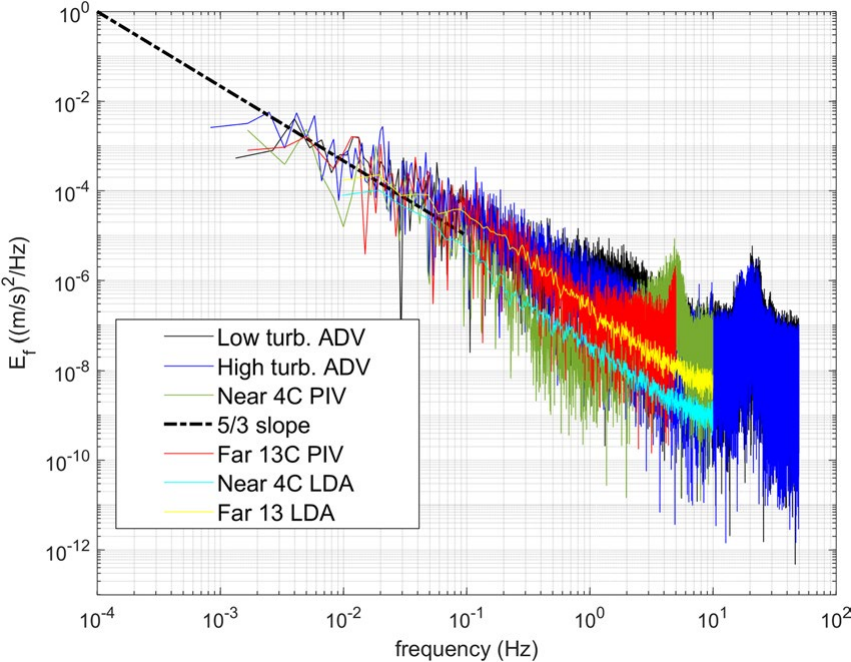
Energy spectra from PIV at LDA crossing for different optical turbulence strengths are consistent with spectra calculated from corresponding LDA data. The data are also consistent with spectra from velocity data collected with the Vectrino Profiler Acoustic Doppler Velocimeter (ADV) during a previous study for comparable turbulence strengths^6^.

To further investigate the frequency distribution in the signal, energy spectra were calculated from the velocity time series collected with the PIV at the location of the LDA crossing, as well as the LDA signal (Fig. 8). The energy spectra from PIV for low (Nearfield, ΔT = 4 °C) and high (Farfield, ΔT = 13 °C) optical turbulence are consistent with spectra calculated from LDA data. The data are also consistent with spectra from velocity data collected with the Vectrino Profiler Acoustic Doppler Velocimeter (ADV) during a previous study for comparable turbulence strength^6^.

As described in detail in^6^, time series from ADV or PIV data, as reported here, are not well suited for the calculation of the turbulent kinetic energy dissipation rate ε in this tank. The inertial subrange, which is instrumental to the estimation of ε, is underresolved in PIV time series data. These data are collected at sampling frequencies less than what would be required to properly resolve the inertial subrange (ideally 50–100 Hz sampling frequency or higher), due to limitations in fire rate of typical PIV lasers. Furthermore, due to the absence of an imposed mean flow in the tank, the mean of the vertical velocity is close to zero, as illustrated in Figs. 3 and 5. This makes the assumption of Taylor’s Frozen Turbulence Hypothesis, which is needed to convert from frequency to wavenumber space for the subsequent estimation of ε, not well suited for this setup. When ε is estimated from these data, as reported in^6^, neither time series from ADV nor PIV, are able to distinguish well between the respective turbulence regimes. This is reflected in Fig. 8, where the spectra of ADV and PIV for high and low turbulence conditions fall largely on top of each other in the inertial subrange (the region of −5/3 slope). Alternatively, to forgo the need for the Frozen Turbulence Hypothesis, it is possible to estimate ε not from time series data, but using wavenumber spectra directly calculated from the PIV spatial fields. However, due to the limited spatial size and resolution of these fields, on the order of 150–200 points for a horizontal section across a PIV FOV as for example in the velocity fields shown in Fig. 4, this severely underrepresents the lower frequencies, i.e. the larger scales, including the inertial subrange. This lack of resolution of the larger turbulent scales is simply a limitation of the size of the FOV and cannot be mitigated by ensemble averaging over realizations at different times. In^6^, we use a combination of ADV time series, PIV spatial maps, and numerical model, to guide our estimates of ε for the respective turbulence strengths. The values are then estimated at around ε = 4·10^−7^ W/kg for the high convective turbulence conditions, and at ε = 3·10^−8^ W/kg for the low convective turbulence conditions. When we apply the Frozen Turbulence Hypothesis to the LDA time series data reported in this paper, which unlike the PIV, show a clear distinction between the regimes in a region of the spectrum not overly influenced by noise, we can estimate ε by extending the spectra into the inertial subrange with guidance from the PIV spectra. The estimated values are consistent with those reported in^6^, at ε = 7·10^−7^ W/kg for high, and ε = 1·10^−8^ W/kg for low convective turbulence. In^6^, it is shown that measurements of temperature and subsequent estimation of temperature variance dissipation rates χ, via the calculation of temperature gradient spectra, allow a better distinction between the respective convective regimes. The turbulence regimes in the tank are characterized by the respective strength of the convective forces, as described by the Rayleigh number (the ratio of convective to viscous-diffusive terms, see also 1.), while the Reynolds number (the ratio of inertial to viscous terms and a classical measure of turbulence strength) remains low to moderate at O(5000) for both regimes. This is reflected in the relatively benign flow velocities in the tank, reaching maximum speeds on the order of 2 cm/s for the strongest convection and similar values for the lower convection regime. The interested reader is referred to^6,13^ which present an in-depth characterization of the flow in the SiTTE tank in terms of turbulence characteristics and optical turbulence conditions, respectively.

We note that the Kolmogorov microscale of the flow at both convective turbulence strengths is estimated to be on the order of O(2 mm). Thus, an interrogation area of a 64 × 64 pixel size, as may be needed under high optical turbulence conditions, will not be able to capture the smaller turbulent eddies. Here, 64 pixels corresponds to about 6.6 mm, - as opposed to 1.6 mm for 16 pixels. Nevertheless, the spectrum from the times series of the PIV measurements for the high optical turbulence case (Farfield, ΔT = 13 °C) follows the −5/3 slope of the Kolmogorov turbulence spectrum and is consistent with the spectrum from the corresponding LDA measurement, thus capturing the main turbulence characteristics of the flow.

**Figure 9:**
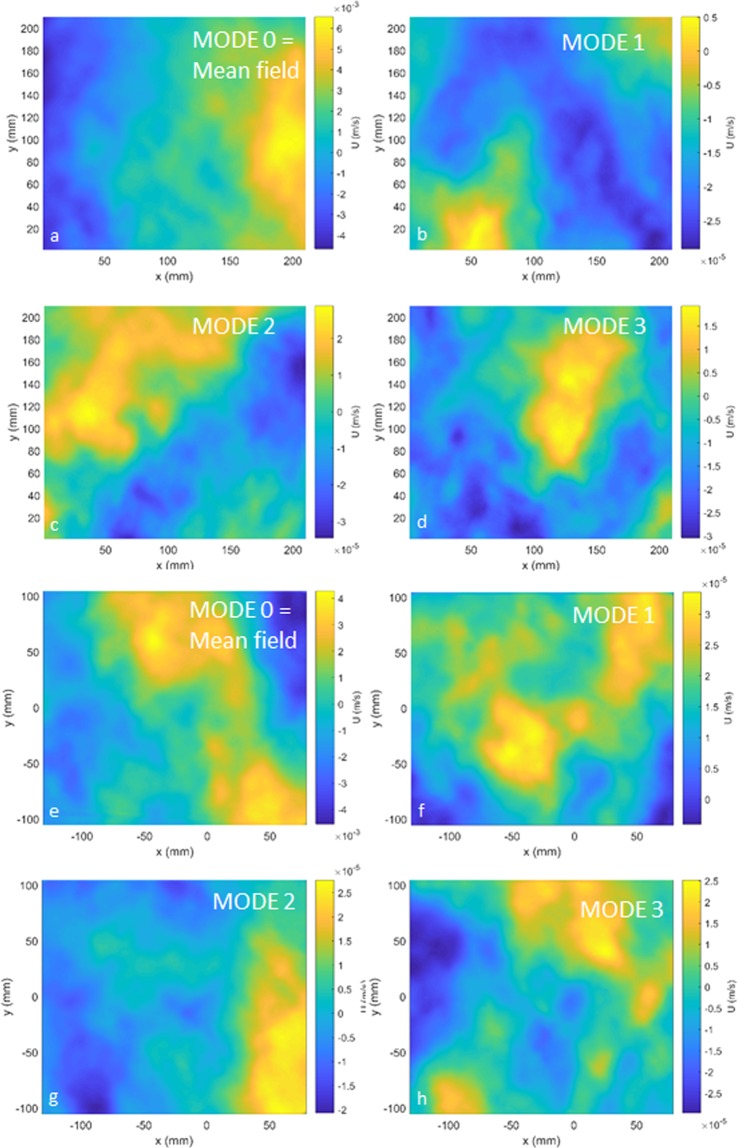
Modes of POD showing the scales of dominant two-dimensional features in the flow. Top 4 panels (**a**–**d**) Short optical path, high turbulence (Nearfield, ΔT = 13 °C), first four modes, where zero modes corresponds to the mean field. Bottom 4 panels (**e**–**h**) Long optical path, high turbulence (Farfield, ΔT = 13 °C), first four modes, where zero modes corresponds to the mean field. Note that the scales of variability are similar for both cases.

To look more in depth at the velocity signal from PIV, we performed a Proper Orthogonal Decomposition (POD). POD is a Principal Component Analysis introduced for turbulence by^14^ and used for Rayleigh-Bénard convection by^15^. The mathematical details are beyond the scope of this paper, but can be found in^16^. POD can describe the scale of the dominant flow features and identify the energy-containing modes. For the Nearfield, high convective turbulence (ΔT = 13 °C) case, modes 1–5 contain 60% of the energy, modes 1–10 contain 72%, and modes 1–20 contain 84% of the signal energy. A similar picture emerges for the long optical path, high turbulence case (Farfield, ΔT = 13 °C), modes 1–5 contain 60%, modes 1–10 contain 73%, and modes 1–20 contain 83% of energy. The latter case had been subject to strong optical turbulence and signal variance was lost in post-processing the PIV images for velocity. Nevertheless, the POD results are similar for both the case of short optical path (low optical turbulence), and the case of long optical path (high optical turbulence) and the scales of variability are comparable (Fig. 9).

**Figure 10:**
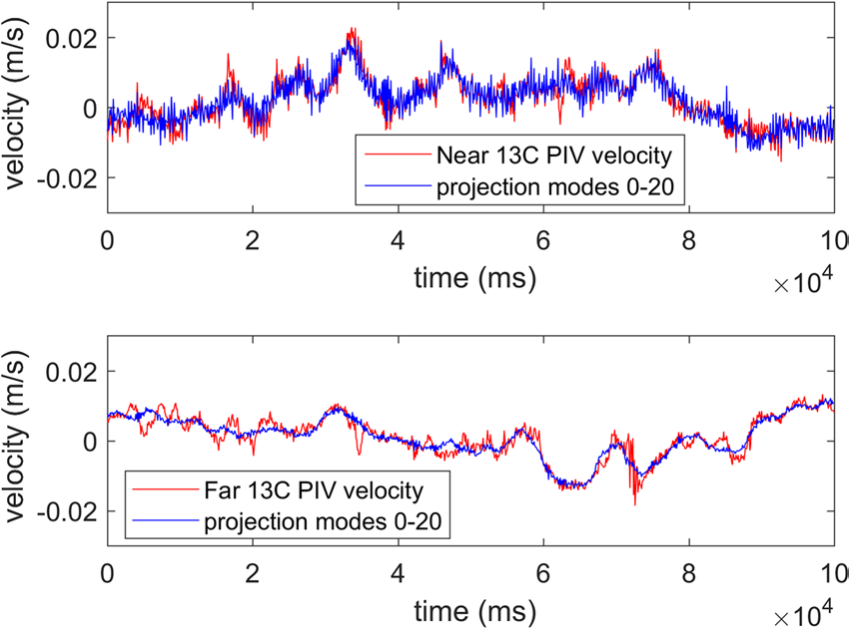
Times series of PIV vertical velocity at LDA crossing compared to time series of POD projection at that point. Top panel is for NearField, i.e., short optical path, at high convective turbulence, ΔT = 13 °C. Bottom panel is for FarField, i.e., long optical path, at high convective turbulence, ΔT = 13 °C. For both, the Nearfield and Farfield at high convective turbulence, the POD projection captures most of the signal energy in the first 20 modes.

The signal recomposed from the first 20 modes of the POD contains most of the signal variability (Fig. 10). While some turbulent variations may be lost with larger interrogation area sizes, the results of the POD reprojections indicate that the majority of the flow energy is captured by the PIV measurements for all cases.

## Discussion

Particle image velocimetry (PIV) is a well-established tool to collect high-resolution velocity and turbulence data in the laboratory. With the increasing interest in using PIV in the oceanographic field setting, the effect of strong density gradients on optical measurement methods can no longer be neglected. We observed a marked influence of optical turbulence on particle imaging in PIV and investigated this effect for various path lengths (0.5 m to 2 m) and optical turbulence strengths. The results indicate that optical turbulence can affect PIV measurements and the impact depends on the strength of the optical turbulence and path length. The effect can be at least partially mitigated in post-processing by using signal appropriate post-processing techniques when inferring velocities from PIV data. Optical turbulence can lead to excessive noise in the PIV velocity signal and while this noise can be removed in post-processing by choosing an appropriate interrogation area size, this noise removal reduces the variance in the signal and may affect the estimation of turbulence parameters. The use of a reference velocity measurement is thus of critical importance when interpreting PIV measurements collected in the presence of optical turbulence. Nevertheless, despite the loss of signal variance, the energy spectra calculated from PIV velocities subject to strong optical turbulence were consistent with spectra calculated from a reference velocity measurement and appear to capture the main turbulent characteristics of the flow. Similarly, results from a principal component analysis, POD, are similar for signals measured under comparable conditions and subject to low and high optical turbulence, respectively.

The impact of optical turbulence on PIV measurements is not only of importance in the case of Rayleigh-Bénard convection as reported here, but can similarly be applied to topics of wide-ranging interest, such as for example, PIV measurements of flow velocities under an ice-covered water surface or in buoyancy-driven currents such as fresh-water lenses or river outflow plumes where density changes due to temperature or salinity are pronounced and optical turbulence effects have been documented. Optimizing measurement and processing techniques under these conditions of optical turbulence can thus be of critical importance to the study of air-sea fluxes and environmental flows.

## Methods

**Figure 11:**
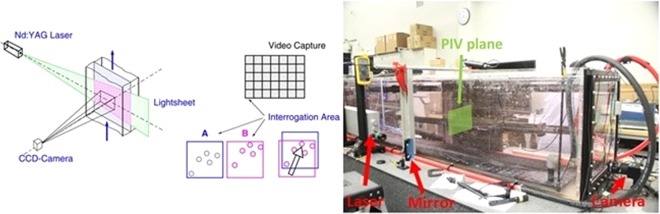
Laboratory tank at NRLSSC. The schematic on the left illustrates the concept of PIV measurements (image source Dantec Dynamics, Inc.). The photo on the right shows the setup of the Particle Image Velocimetry (PIV) system for a measurement plane with a path length of about 1 m. The mirror is set up to direct the light sheet (green laser, wavelength 532 nm) into the tank at a 90 degree angle to illuminate a cross section. In the photo, the camera is looking on the bottom boundary, whereas for the experiments presented in this paper, the camera was positioned to look at the center height of the tank (after^6^).

The PIV set up is a state-of-the-art system by Dantec Dynamics Inc. designed to measure two-dimensional cross-sectional planes of the convective flow in the SiTTE tank. In PIV, a laser sheet illuminates a flow seeded with small particles while a CCD camera records image pairs at high frequency. Correlations between particles in successive images, i.e., how much the particles have moved between frames, are used to infer flow velocities. Figure 11 illustrates the basic PIV concept and shows the experimental setup at SiTTE: the laser, the mirror directing the laser sheet into the tank at a 90 degree angle to the tank side wall to make a cross section, as well as the CCD camera. The laser is a Litron ND:Yag laser, dual cavity, 135 mJ per cavity, with a 15 Hz maximum frequency per cavity. The laser light used to generate the PIV light sheet has wavelength of 532 nm. The water in the tank was seeded with silver-coated glass particles (10 µm diameter) at a seeding density of 0.06 particles per pixel. The average particle size image was approximately 2.3 pixels. For the lower velocities encountered at low Rayleigh numbers and thus lower convective and optical turbulence (ΔT = 4 °C and ΔT = 8 °C), the flow measurement was time-resolved (i.e., successive images in the ensemble were correlated in time) and the sampling frequency was set to 20 Hz for the collection of single frame images. For the high Rayleigh number, and thus high convective turbulence cases (ΔT = 13 °C), the sampling frequency was set to 10 Hz, with an interval of 30 ms between laser pulses to record double frame image pairs. The images were collected with an image size of 2048 × 2048 pixel. The spatial resolution of the derived velocity field varies depending on the post-processing algorithm, but is on the order of 1–2 mm per interrogation area.

Velocities from the PIV measurements were calculated using the algorithms provided within Dantec’s Dynamic Studio, including Adaptive Correlation, which holds the final interrogation area fixed and Adaptive PIV, which adjusts interrogation area size and shape locally to optimize for seeding density and velocity gradient variations. The principle of Adaptive Correlation is an iterative procedure, where a shifted window is used to increase the correlation peak. The size of the interrogation area is iteratively decreased to fit the particle pattern between the two time steps with the final interrogation area size fixed^17^. For the Adaptive Correlation processing, an initial interrogation area size of 64 × 64 pixels was used with two iterative steps and final interrogation area size of 16 × 16 pixels. A minimum correlation peak height ratio value of 1.2 was required for validating vectors. Additionally, a 5 × 5 local median validation was employed, however, rejected vectors were not substituted and no smoothing applied (in order to accurately assess the effect of optical turbulence). For Adaptive PIV processing, the interrogation areas were allowed to vary from 16 to 64 pixels in the x and y directions. The particle adaptivity was set with a minimum number of five and desired number of ten particles per interrogation area. For gradient adaptivity, the absolute value of each component of the velocity gradient, was limited to 0.1 while the total magnitude of the gradients (square root of the sum of the squares) was limited to 0.2. The same validation criteria were applied to Adaptive PIV as described for Adaptive Correlation.

**Figure 12:**
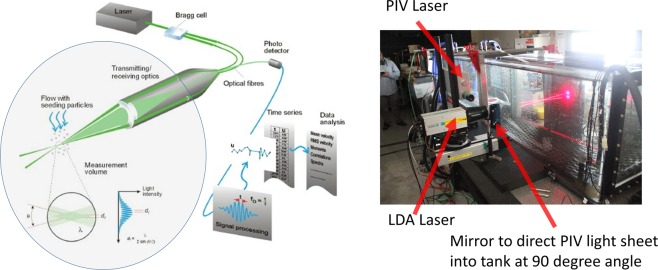
Set up for experiments presented in this paper. The schematic on the left illustrates the concept of Laser Doppler Anemometer (LDA) measurements (image source Dantec Dynamics, Inc.). The photo on the right shows the setup of the LDA system at SiTTE to provide a reference measurement, as well as the PIV system shown in Fig. 11. The LDA system is positioned so that the intersection of the red laser beams (wavelength 660 nm) is in same plane as the PIV laser sheet.

A Laser Doppler Anemometer (LDA) system by Dantec Dynamics Inc. was used to provide an independent reference measurement of velocity. The LDA provides a point measurement of velocity and was positioned in such a way that the measurement point was in plane with the PIV laser sheet. The system was oriented so that the vertical velocities from PIV and LDA were aligned. The LDA system measurements were taken through the side of the tank perpendicular to the camera window and the same path length of 0.3 m was maintained for all measurements (while the path length of PIV varied). Figure 12 shows the concept of LDA and the measurement setup with the intersection of the red laser beams indicating the LDA measurement point. Post-processing was performed by using Dantec Dynamics, Inc. proprietary LDA software.

While the LDA, as an optical measurement, is also susceptible to optical turbulence effects, these effects are considered negligible for the set up presented here due to the short optical path (Rytov variance σ^2^ < 0.01). This is based on the assumption that the influence from the optical turbulence in the tank should be the same for both probing beams of the LDA, and should not affect the Doppler frequency shift, and thus the pattern from which the particle velocities are derived. This might not be the case if the beams were spatially separated by a large amount, or extreme optical turbulence and velocities were present. In these extreme cases, the frequency shift of each beam could be potentially affected differently and this would have to be taken into consideration.

The choice of LDA as reference measurement was made due to the non-intrusive nature of the LDA method and the possibility to align the LDA point measurement with high accuracy within the PIV light sheet.
